# Generation of digital patients for the simulation of tuberculosis with UISS-TB

**DOI:** 10.1186/s12859-020-03776-z

**Published:** 2020-12-14

**Authors:** Miguel A. Juárez, Marzio Pennisi, Giulia Russo, Dimitrios Kiagias, Cristina Curreli, Marco Viceconti, Francesco Pappalardo

**Affiliations:** 1 School of Mathematics and Statistics, University of Sheffield, Sheffield, S3 7RH UK; 2 Computer Science Institute, DiSIT, University of Eastern Piedmont, Alessandria, Italy; 3 Department of Drug Sciences, University of Catania, Catania, Italy; 4 Department of Industrial Engineering, University of Bologna, Bologna, Italy

**Keywords:** Agent based model, In silico patient, Sequential sampling, Tuberculosis

## Abstract

**Background:**

The STriTuVaD project, funded by Horizon 2020, aims to test through a Phase IIb clinical trial one of the most advanced therapeutic vaccines against tuberculosis. As part of this initiative, we have developed a strategy for generating in silico patients consistent with target population characteristics, which can then be used in combination with in vivo data on an augmented clinical trial.

**Results:**

One of the most challenging tasks for using virtual patients is developing a methodology to reproduce biological diversity of the target population, ie, providing an appropriate strategy for generating libraries of digital patients. This has been achieved through the creation of the initial immune system repertoire in a stochastic way, and through the identification of a vector of features that combines both biological and pathophysiological parameters that personalise the digital patient to reproduce the physiology and the pathophysiology of the subject.

**Conclusions:**

We propose a sequential approach to sampling from the joint features population distribution in order to create a cohort of virtual patients with some specific characteristics, resembling the recruitment process for the target clinical trial, which then can be used for augmenting the information from the physical the trial to help reduce its size and duration.

## Background

It is estimated that one quarter of the world population is infected with (TB). Although the disease is preventable and treatable, about one and a half million people die annually from it, effectively placing TB as the first infectious cause of death. Due to person to person infection and treatment mismanagement, (MDR) TB continues to emerge, increasing the complexity in treatment and thus potentially worsening the transmission rate. There is a growing awareness that TB can be effectively fought only working globally, starting from countries like India, where the infection is endemic [1].

Once a person is diagnosed with TB, one of the most critical issues is the duration of the therapy, because of the high costs involved, the increased chances of non-compliance (which increase the probability of developing an MDR strain), and the time the patient is still infectious to others. One exciting possibility to shorten the duration of the therapy are novel host-reaction therapies (HRT), as an adjuvant for antibiotic therapy. Typical endpoints in the clinical trials for HRTs are time to sputum culture conversion, and incidence of recurrence. While for the first it is in some cases possible to have a statistically powered evidence for efficacy in a phase II clinical trial, recurrence almost always requires a phase III clinical trial with thousands of patients involved, and huge costs.

The in silico trials for tuberculosis vaccine development (STriTuVaD) project is an EU funded, multidisciplinary consortium testing the RUTI vaccine in a Phase IIb clinical trial. RUTI^®^ antitubercular vaccine, provided by Archivel Farma S.L, is a polyantigenic liposomal vaccine containing fragments of Mycobacterium tuberculosis cells, currently being developed as therapeutic vaccine in patients with pulmonary tuberculosis. The vaccine, shown to be one of the most advanced therapeutic vaccines against drug sensitive TB and MDR-TB, has already been studied in healthy volunteers and for the prevention of active TB in patients with latent TB [2].

To help in this development, we extend Universal Immune System Simulator (UISS) [3, 4] to include the relevant determinants of such clinical trial, we establish its predictive accuracy against the individual patients recruited in the trial, use it to generate digital patients, predict their response to the host-reaction therapy being tested, and combine them to the observations made on physical patients using a new in silico-augmented clinical trial approach that uses a Bayesian adaptive design. This approach, where found effective could drastically reduce the cost of innovation in this critical sector of public healthcare.

To reproduce biological the diversity of the subjects to be simulated, an appropriate strategy for the generation of libraries of digital patients is developed by identifying a vector of features involving both biological and pathophysiological parameters, facilitating the personalisation of the digital patient.

In this paper we sketch the strategy we adopt to generate the cohort of digital patients, and show some preliminary results about the dynamics of TB on a subset of these patients. First, we briefly describe UISS and its extension to TB.

### Extending UISS to track TB

We will briefly describe here the UISS computational framework and its extension to model tuberculosis, UISS-TB. The interested reader can find more detail in [5].

UISS is a multi-agent framework for the simulation of the immune system dynamics that can be extended to track specific diseases and related treatments. Unlike classical top-down approaches, where mean behaviours are modelled through systems of differential equations [6–8], agent based models and multi-agent systems track individual entities. It is the interactions between these entities that can give rise to global nonlinear behaviours. UISS has been developed as a multi-scale computer simulator of the immune system, as it takes into account both cellular and molecular entities and processes.

UISS has a proven track record, for instance it has been used for modelling the effects of a vaccine against the onset of mammary carcinoma [9, 10] and consequent lung metastases [11]; for the initial stages of atherosclerosis [12], for melanoma [3]; more recently, in the study of multiple sclerosis [4, 13] and for testing the efficacy of citrus-derived adjuvants for influenza vaccines and human papilloma virus [14, 15]. For its use within STriTuVaD, we have extended UISS to include TB dynamics along with the artificial immunity induced by vaccination strategies as presented in [5].

In order to depict individuals, a vector of features comprising biological and pathophysiological parameters has been identified. The list of parameters, their relative range and units are displayed in Table  1.

**Table 1:** Vector of 22 features for individualising virtual patients

Feature	Range	Units	Type	Notation
Bacterial load in sputum	[0–10000]	CFU	D	MtbSputum
MTB virulence	[0–1]	—	C	strain
CD4 T cell type 1	[0–100]	$\hbox {cells}/\upmu \hbox {L}$	D	Th1
CD4 T cell type 2	[0–100]	$\hbox {cells}/\upmu \hbox {L}$	D	Th2
Specific IgG titers	[0–512]	IgG titer	C	IGg
CD8 T cell	[0–1134]	$\hbox {cells}/\upmu \hbox {L}$	D	TC
Interleukin 1	[0–235]	pg/mL	C	IL1
Interleukin 2	[0–894]	pg/mL	C	IL2
Interleukin 10	[0–516]	pg/mL	C	IL10
Interleukin 12	[0–495]	pg/mL	C	IL12
Interleukin 17	[0–704]	pg/mL	C	IL17
Interleukin 23	[0–800]	pg/mL	C	IL23
Interferon-$\alpha$	[0–148.4]	pg/mL	C	IFN1A
Interferon-$\beta$	[0–206]	pg/mL	C	IFN1B
Interferon-$\gamma$	[0–268.2]	pg/mL	C	IFng
TNF-	[0–49.4]	pg/mL	C	TFN
LXA4	[0–3]	ng/mL	C	LXA4
PGE2	[0–2.1]	ng/mL	C	pgE2
Vitamin D	[25–80]	ng/mL	C	VD
Regulatory T cells	[0–200]	$\hbox {cells}/\upmu \hbox {L}$	D	Treg
Age	[10–80]	years	D	Age
Body Mass Index	[18.5–35]	$\hbox {kg}/\hbox {m}^{2}$	C	BMI

Type (Discrete or Continuous), relative range with units of measure and notation used in the paper

## Methods

In order to create an in silico patient, one needs to provide a single value for each feature. These values could be taken from individual physical patients; however, if a cohort of digital patients is to be produced, one should have a mechanism for producing as many different input vectors as needed, that are biological/physiological plausible. Formally, this requires the characterisation of the joint distribution of the inputs in the population. We have compiled typical values and standard deviations for each feature, providing a way to generate plausible values for each component at a time. Proceeding in this way would neglect the biological correlations between features and thus would not guarantee a physiologically plausible input vector. Hence, we must take into account these correlations. Given that we have 22 input variables, we should specify $22 \times 21/2 = 231$ correlations. Using relevant literature [16, and references therein] and expert opinion, we have qualified these correlations, determining that all correlations are positive, but the correlation of IL-10 with the rest of the features.

### Formalising in silico profile generation

In theory, one could elicit the joint distribution of the features vector, i.e. describe mathematically how each feature relates to the others in a space of 22 dimensions; but this would be not only extremely difficult, but also time consuming and data demanding. Our approach is to rely on current mathematical biology consensus and use a Gaussian to represent the population distribution. The additional advantage of using this approach will be discussed in the next section.

Formally, we say that the vector ${\varvec{f}}$ = $\left\{ {f_{1},\dots ,f_{d}}\right\}$ follows a *d*-variate Gaussian distribution with joint probability density function,

$$\begin{aligned} \hbox{N}_{d} ({\varvec{f}} | \boldsymbol{\mu }, {\Sigma }) = \frac{|\Sigma |^{-1/2}}{(2 \pi )^{d/2}} \exp \left[ {- \frac{1}{2} \left( {{\varvec{f}} - \boldsymbol{\mu }}\right) ' \Sigma ^{-1} \left( {{\varvec{f}} - \boldsymbol{\mu }}\right) }\right] , \end{aligned}$$

with mean $\boldsymbol{\mu } = \left\{ {\mu _{1},\dots ,\mu _{d}}\right\}$ and covariance matrix,

$$\begin{aligned} \Sigma = \left( {\begin{matrix}\sigma ^2_{1} &{} \sigma _{12} &{} \ldots &{} \sigma _{1d} \\ \sigma _{21} &{} \sigma ^2_{2} &{} \ldots &{} \sigma _{2d} \\ \vdots &{} \vdots &{} \ddots &{} \vdots \\ \sigma _{d1} &{} \sigma _{d2} &{} \ldots &{} \sigma ^2_{d} \end{matrix}}\right) \,, \end{aligned}$$

where,

$$\begin{aligned} {{\,\mathrm{Cov}\,}}\left( {x_i, x_j}\right) = \sigma _{ij} \, \text {related to the correlations by} \, {{\,\mathrm{Cor}\,}}\left( {x_i, x_j}\right) = \rho _{ij} = \frac{\sigma _{ij}}{\sqrt{\sigma ^2_i\sigma ^2_j}}\,. \end{aligned}$$

So, if we are able to elicit a measure of correlation between two inputs, we can calculate their covariance.

The elements in the diagonal, $\sigma ^2_i$ are the marginal variances of each element, $f_i$, and $\mu _i$ the corresponding marginal mean. As mentioned above, we already have compiled a list with these values, so we have elicited values for $\boldsymbol{\mu }$ and the diagonal elements of $\Sigma$, $\sigma ^2_i$.

### Cohort generation

Once $\boldsymbol{\mu }$ and $\Sigma$ have been elicited, generating an in silico profile is a relatively trivial task: one must sample a point in the 22-dimensional space, consistent with $\hbox{N}_{22} ({\varvec{f}} | {\boldsymbol{\mu }}, {\Sigma })$. However, we can exploit the properties of the Gaussian distribution to produce a cohort consistent with some specific characteristics. Say, for instance, that our target population has a particular range of BL, we would like then to produce digital patients consistent with that specific profile. Formally, let $f_1$ represent BL and ${\varvec{f}} _{-1} = \left\{ {f_{2},\dots ,f_{22}}\right\}$, the rest of the features; we would like to sample from $\hbox{N}_{21} ({\varvec{f}} _{-1} | {f_1, \boldsymbol{\mu }}, {\Sigma })$, ie the conditional distribution of the rest of the features, given that BL has a specific value. This is a standard procedure, which can be readily implemented.

We can go further and sort the list of features according to either their importance in determining the profile of a patient, or to the precision of their elicited mean, variance and covariance, and then proceed to sample from the conditional distributions. In general, let ${\varvec{f}} _s$ denote the vector of features with pre-specified values, so that ${\varvec{f}} = \left\{ {{\varvec{f}} _s, {\varvec{f}} _r}\right\}$, ${\varvec{f}} _s \in {\mathbb{R}}^{d-q}$, where ${\varvec{f}} _r \in {\mathbb{R}}^q$ is the vector of free features.

The conditional distribution, $p ({{\varvec{f}} _r}| {{\varvec{f}} _s = {\varvec{a}}})= \hbox{N}_{q} ({\varvec{f}} _r | {\boldsymbol{\nu }}, {\Omega })$ with

$$\begin{aligned} \boldsymbol{\nu } = \boldsymbol{\mu } _r + \Sigma _{rs} \Sigma _{ss}^{-1} ({\varvec{a}} - \boldsymbol{\mu } _s) \quad \text {and} \quad \Omega = \Sigma _{rr} - \Sigma _{rs} \Sigma _{ss}^{-1} \Sigma _{sr}, \end{aligned}$$

where

$$\begin{aligned} \Sigma = \left( {\begin{matrix} \Sigma _{ss} &{} \Sigma _{sr} \\ \Sigma _{rs} &{} \Sigma _{rr} \end{matrix}}\right) \quad \text {with sizes} \quad \left( {\begin{matrix} (d-q) \times (d-q) &{} (d-q) \times q \\ q \times (d-q) &{} q \times q \end{matrix}}\right) . \end{aligned}$$

$\Omega$ the Schur complement of $\Sigma _{rr}$ in $\Sigma$. Judicious choice of ${\varvec{f}} _s$ and ${\varvec{f}} _r$ enables sampling sequentially, e.g. from least to most important feature.

## Results

We created an R script [17] for the generation of digital patents, available from the corresponding author upon request. We report results from three groups of 15 patients with different profiles, each with fixed (Age, BMI and MtbSputum) to roughly represent different profiles in the population and initial bacterial load. Profile 1 has (35, 21.4, 15), Profile 2 (45, 28.2, 502), and Profile 3 (55, 31.8, 910), the full set of values can be obtained from the Additional file 1. These can be used as input to the UISS-TB web interface, available from www.strituvad.eu (accessed on 28/07/20), by selecting the Tuberculosis disease model, hence accessible to any user with a conventional computer and access to the internet.

The GUI panel displays default values and admissible ranges for the vector of features parameters. Once the specific vector of features is completed, the user can click on the Submit button and a unique identification simulation number is assigned. The user can check the simulation status by clicking on the check status button, after selecting the appropriate simulation id. When the simulation is complete, the user can visualise results of immune system dynamics. In our case, the progression of each patient was simulated 50 times for 1 year, with levels of the various species recorded every 600 seconds. The data from each patient requires roughly 100 MB of disk storage.

**Fig. 1:**
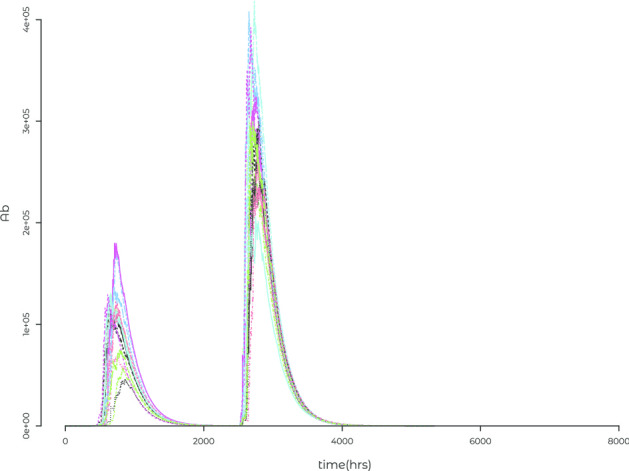
Profile 1 antibodies count. Time traces of the antibodies count for the 15 virtual patients in Profile 1, using only one out of the 50 simulations

**Fig. 2:**
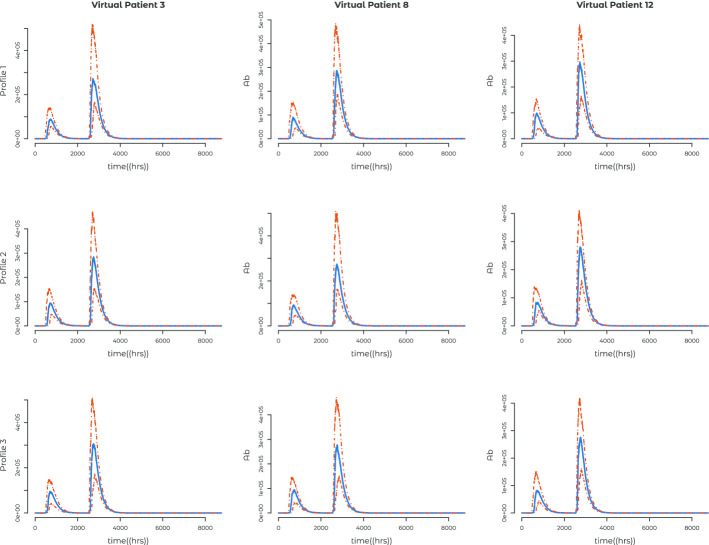
Average antibodies count. Time traces of the average antibodies count for a sample of 3 virtual patients from each profile. The count has a main peak roughly at 4.5 hrs regardless of the profile

**Fig. 3:**
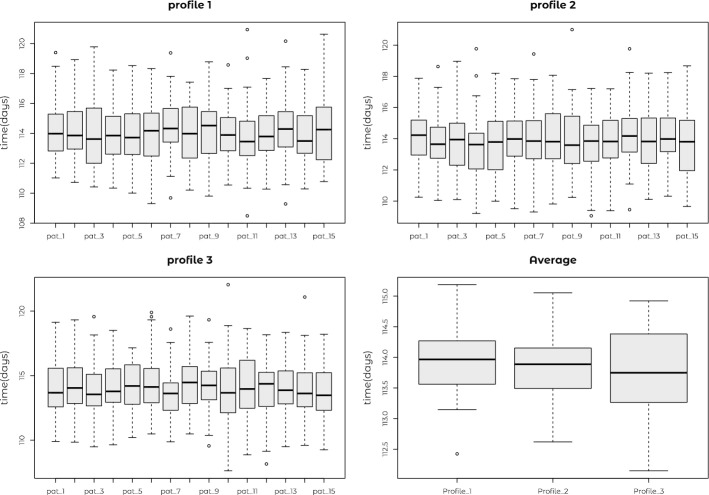
Time at peak. Distribution of time at peak antibodies count by patient and profile and the distribution of the average by profile

We use the total (Ab) to exemplify some characterisation of the output; e.g. Fig. 1 shows the total Ab count for one simulation of the 15 patients in Profile 1. In order to characterise the mean behaviour, we average the 50 repetitions per patient. Figure 2 depicts the median and quartiles for a selection of patients (columns) for each profile (rows). It is clear there is an increased variability around the main and secondary peaks; while levels consistently fall back to nought after roughly 16 days (3500 h). The distribution of time at the peak level is illustrated in Fig. 3, it occurs consistently within 112–116 days for all profiles, while Profile 3 shows a slightly increased variability.

## Conclusions

UISS-TB is a state-of-the-art agent based model capable of tracking the dynamics of TB infection in humans. Individual digital patients are defined by a vector features, known to be fundamental in TB infection dynamics and normally measured clinically, hence often readily available.

## Discussion

In order to produce virtual cohorts of patients, we propose a sequential approach based on a characterisation of the distribution of these features in the population of interest; the approach allows to fix any combination of features, enabling mimicking patient selection criteria, thus yielding a method for setting up augmented in silico clinical trials.

## Supplementary information

**Additional file 1:** Profile traces.

## Data Availability

The datasets generated and analysed during the current study are not publicly available due to size restrictions but are available from the corresponding author on reasonable request.

## Abbreviations

Ab: Antibody count
MDR: Multi-drug resistant
STriTuVaD: In silico trials for tuberculosis vaccine development
TB: Tuberculosis
UISS: Universal Immune System Simulator

## References

[1] WHO: Global tuberculosis report (2019).

[2] Prabowo SA, Painter H, Zelmer A, Smith SG, Seifert K, Amat M, Cardona P-J, Fletcher HA (2019). RUTI vaccination enhances inhibition of mycobacterial growth ex vivo and induces a shift of monocyte phenotype in mice. Front Immunol.

[3] Pappalardo F, Forero IM, Pennisi M, Palazon A, Melero I, Motta S (2011). SimB16: modeling induced immune system response against B16-melanoma. PLoS ONE.

[4] Pennisi M, Russo G, Motta S, Pappalardo F (2015). Agent based modeling of the effects of potential treatments over the blood brain barrier in multiple sclerosis. J Immunol Methods.

[5] Pennisi M, Russo G, Sgroi G, Bonaccorso A, Parasiliti Palumbo GA, Mitra DK, Walker KB, Cardona P-J, Amat M, Viceconti M, Pappalardo F (2019). Predicting the artificial immunity induced by RUTI® vaccine against tuberculosis using universal immune system simulator (UISS). BMC Bioinform..

[6] Ragusa MA, Russo G (2016). ODEs approaches in modeling fibrosis: comment on “Towards a unified approach in the modeling of fibrosis: a review with research perspectives” by Martine Ben Amar and Carlo Bianca. Phys Life Rev.

[7] Castiglione F, Pappalardo F, Bianca C, Russo G, Motta S (2014). Modeling biology spanning different scales: an open challenge. BioMed Res Int.

[8] Pappalardo F, Pennisi M, Ricupito A, Topputo F, Bellone M (2014). Induction of T-cell memory by a dendritic cell vaccine: a computational model. Bioinformatics.

[9] Pappalardo F, Motta S, Lollini P-L, Mastriani E (2006). Analysis of vaccine’s schedules using models. Cell Immunol.

[10] Palladini A, Nicoletti G, Pappalardo F, Murgo A, Grosso V, Stivani V, Ianzano ML, Antognoli A, Croci S, Landuzzi L, De Giovanni C, Nanni P, Motta S, Lollini P-L (2010). In silico modeling and in vivo efficacy of cancer-preventive vaccinations. Cancer Res.

[11] Pennisi M, Pappalardo F, Palladini A, Nicoletti G, Nanni P, Lollini P-L, Motta S (2010). Modeling the competition between lung metastases and the immune system using agents. BMC Bioinform.

[12] Pappalardo F, Musumeci S, Motta S (2008). Modeling immune system control of atherogenesis. Bioinformatics.

[13] Pappalardo F, Russo G, Maimone D, Pennisi M, Sgroi G, Alessandro G, Pappalardo F, Russo G, Pennisi M, Sgroi G, Alessandro G, Palumbo P, Motta S, Maimone D. Agent based modeling of relapsing multiple sclerosis: a possible approach to predict treatment outcome. In IEEE international conference on bioinformatics and biomedicine (BIBM). 2018;1380–5.

[14] Pappalardo F, Fichera E, Paparone N, Lombardo A, Pennisi M, Russo G, Leotta M, Pappalardo F, Pedretti A, De Fiore F, Motta S (2016). A computational model to predict the immune system activation by citrus-derived vaccine adjuvants. Bioinformatics.

[15] Pennisi M, Russo G, Ravalli S, Pappalardo F (2017). Combining agent based-models and virtual screening techniques to predict the best citrus-derived vaccine adjuvants against human papilloma virus. BMC Bioinform.

[16] Mayer-Barber KD, Andrade BB, Oland SD, Amaral EP, Barber DL, Gonzales J, Derrick SC, Shi R, Kumar NP, Wei W, Yuan X, Zhang G, Cai Y, Babu S, Catalfamo M, Salazar AM, Via LE, Barry CE, Sher A (2014). Host-directed therapy of tuberculosis based on interleukin-1 and type I interferon crosstalk. Nature.

[17] R Core Team: R: a language and environment for statistical computing. R Foundation for Statistical Computing, Vienna, Austria (2020). Version 4.0.2.

[18] Pennisi M, Juarez MA, Russo G, Viceconti M, Pappalardo F. Generation of digital patients for the simulation of tuberculosis with UISS-TB. In: 2019 IEEE international conference on bioinformatics and biomedicine (BIBM), 2019;2163–2167. 10.1186/s12859-020-03776-z PMC7733699 33308156

